# A flexible framework for anomaly Detection via dimensionality reduction

**DOI:** 10.1007/s00521-021-05839-5

**Published:** 2021-03-11

**Authors:** Alireza Vafaei Sadr, Bruce A. Bassett, M. Kunz

**Affiliations:** 1 Département de Physique Théorique and Center for Astroparticle Physics, University of Geneva, Geneva, Switzerland; 2 Institute for Research in Fundamental Sciences (IPM), P. O. Box 19395-5531, Tehran, Iran; 3 African Institute for Mathematical Sciences, Cape Town, South Africa; 4 South African Radio Astronomy Observatory, University of Cape Town, Cape Town, 7925 South Africa; 5 Mathematics Department, University of Cape Town, Cape Town, 7700 South Africa

**Keywords:** Anomaly detection, Outlier detection, Cluster analysis, Novelty detection

## Abstract

Anomaly detection is challenging, especially for large datasets in high dimensions. Here, we explore a general anomaly detection framework based on dimensionality reduction and unsupervised clustering. DRAMA is released as a general python package that implements the general framework with a wide range of built-in options. This approach identifies the primary prototypes in the data with anomalies detected by their large distances from the prototypes, either in the latent space or in the original, high-dimensional space. DRAMA is tested on a wide variety of simulated and real datasets, in up to 3000 dimensions, and is found to be robust and highly competitive with commonly used anomaly detection algorithms, especially in high dimensions. The flexibility of the DRAMA framework allows for significant optimization once some examples of anomalies are available, making it ideal for online anomaly detection, active learning, and highly unbalanced datasets. Besides, DRAMA naturally provides clustering of outliers for subsequent analysis.

## Introduction

Anomaly and novelty detection is an important area of machine learning research and critical across a spectrum of applications that stretch from humble data cleaning to the discovery of new species or classes of objects. An example of the latter application is provided in astronomy by the LSST^1^ and SKA^2^, the next-generation optical and radio telescopes which are so much more powerful than existing facilities that they are expected to observe completely new types of celestial objects and will generate datasets in the range of 100PB–10EB. Other real-world applications include adverse reaction identification in medicine, fraud detection, terrorism, network attacks, abnormal customer behavior, and even applications in the recent COVID-19 pandemic [13, 27, 43, 53]. The wide variety of potential anomalies has led to the development of a range of proposed anomaly-detection methods.

These include *density-based* methods like local outlier factor (LOF) [8] which compares the local density at a point to the density at that point’s neighbors. *Clustering-based* methods, such as DBSCAN [15], detect outliers that belong to very small clusters or lie far from existing clusters. *Distance-based* methods look for outliers by computing distances between all objects. Algorithms in this class include top-n kNN distance [45] and isolation forest (i-Forest) [36], which uses path length through decision trees to identify anomalies. Another possible classification, proposed by Li et al. split the methods into two general categories, principle-based (white-box) and empirical-based (black-box). There is also the spectrum in between (gray-box) [33].

Using dimensionality reduction algorithms is quite a popular approach especially for high-dimensional datasets like networks [12, 18, 24, 28, 29, 32] and medical scans [34]. Faust et al. suggested a classification approach based on dimensionality reduction can work for anomaly detection. Moreover, deep learning-based algorithms use the depth of their architectures to learn important features in the data which can then be used to search for anomalies based on the features [3, 10, 14, 39, 44]. As one example Sakurada and Yairi [49] proposed using autoencoders for anomaly detection. They evaluated their proposed method versus linear PCA and kernel PCA on simulated and real data. They showed that autoencoders can detect subtle anomalies that linear PCA fails to find. Interested readers are encouraged to read the review papers by Pang et al. [40] and Thudumu et al. [52].

Despite the wide range of approaches, the problem is still one of the most challenging areas of machine learning. The no free lunch (NFL) theorems^3^ imply that no “best” anomaly detection algorithm exists across all possible anomalies, classes, data, and problems. For any algorithm, it is possible to construct anomaly attacks that deceive the algorithm by exploiting the features learned in the process of training the algorithm. How can one build a trap for a new type of animal if one knows nothing at all about that animal?

One might be tempted to try to circumvent this aspect of the NFL theorems by building a very large number of features in the hope that some features will, by chance, be sensitive to the anomalous signal. Unfortunately, significantly increasing the number of features leads to the curse of dimensionality [1]: the performance of most machine learning algorithms deteriorates as the dimensionality of the feature space increases dramatically. The key reasons for the “curse” are that distance measures become less and less informative [6] and feature space volume grows exponentially in higher dimensions. Additionally, anomaly detection is hampered by the fact that lack of training examples means it is difficult or impossible to learn the anomalous features or tune any hyperparameters [16]. As an example, deep neural networks succeed as classifiers precisely because they use a large amount of training data to learn the right features to classify the training data. On the other hand, with few or no examples of the anomalies for the training process, there is no way to train the algorithm to be sensitive to the “right” features that will allow the anomalies to be detected. Likewise, a good algorithm for a given class of anomalies may perform badly simply due to poor hyperparameter settings.

Contrary to the dual challenges posed by the NFL theorems and the curse of dimensionality, humans are relatively good at anomaly detection in the real world and have the ability to learn from a single example. It is therefore reasonable to believe that there exist optimal anomaly detectors for subclasses of anomalies relevant to the real world. Most physically relevant functions are fairly smooth and can be efficiently compressed [35]. This inspires our search for “better” algorithms and is the key context of the present work. Application to the case of relatively smooth functions and real-world anomaly datasets is how our anomaly detection algorithm is judged, which we call the dimensionality reduction anomaly meta-algorithm (DRAMA). DRAMA^4^ is released as a python package^5^.

It is useful in some cases to make a technical distinction between anomalies and novelties. Here, anomaly detection corresponds to the case where both the training and test data contain outliers while “novelty detection” is the case where one has “pure” training data (with no outliers) while the test data may have interesting exceptions one wishes to identify. DRAMA can perform both tasks, but flexibility in the various steps allows us to freely use any examples of known anomalies in the training data to find the optimal hyperparameters with respect to the desired figure of merit, choice of dimensionality reduction, and distance metric.

Comparison of our algorithm, DRAMA, with a large number of existing algorithms, is computationally infeasible. We, therefore, pick two popular general algorithms to benchmark DRAMA against local outlier factor (LOF) [8] and isolation forest (iForest) [36]. Benchmarks are performed both against simulated data and a collection of real-world anomaly datasets. The outline of this paper is as follows: in Sect. 2 DRAMA is outlined while Sect. 3 describes the simulated and experimental datasets and metrics. Results and discussion are presented in Sect. 4. Finally, our conclusion can be found in Sect. 5.

## The DRAMA algorithm

**Fig. 1:**
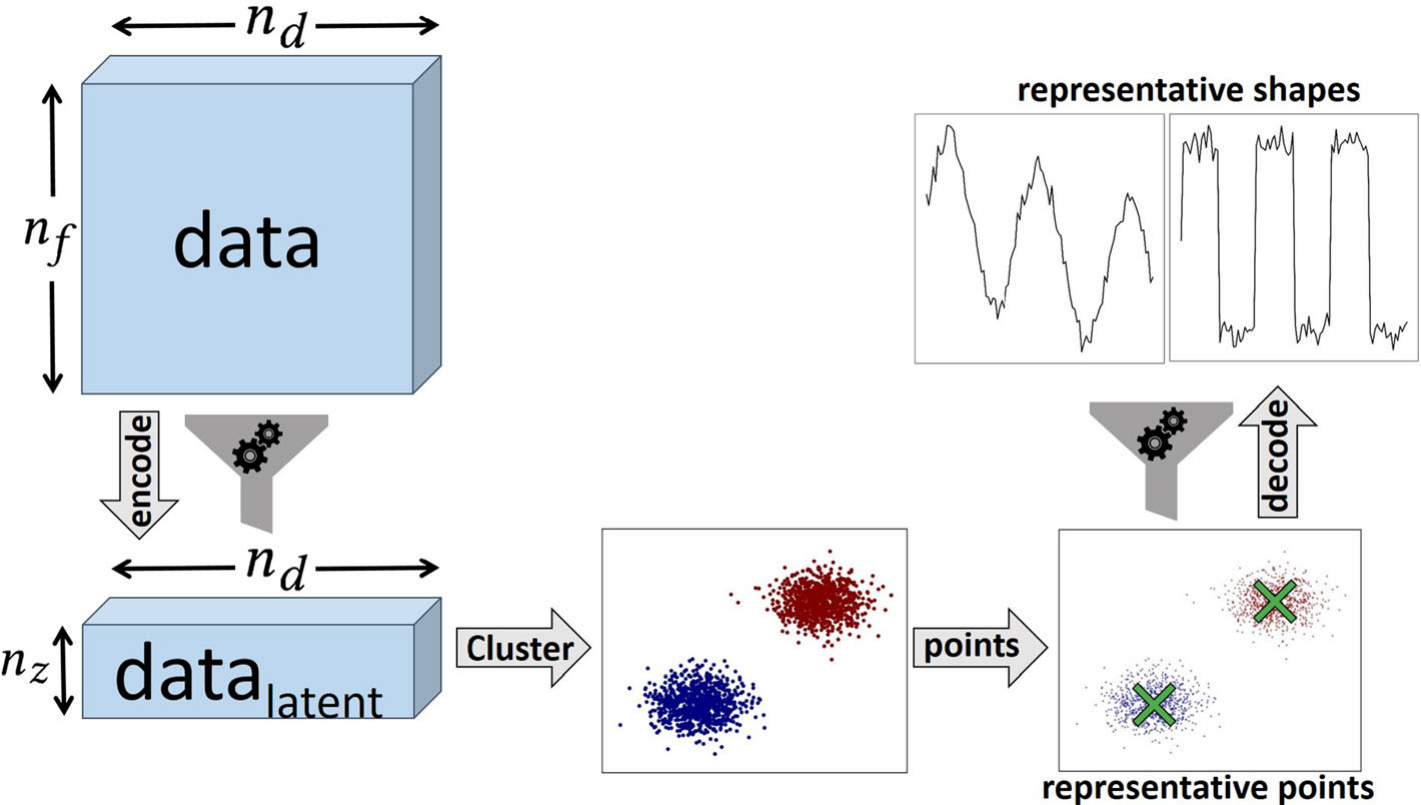
Schematic of the DRAMA framework. Dimensionality reduction (left) is performed on the $n_d$ data points and reduces the number of features from $n_f$ to $n_z$ in the latent space. Clustering then splits the data (here into two clusters). One prototype is extracted for each cluster. Then, the prototypes (main components) are decoded back into the original space (top right). Comparison between the prototypes and test data ranks data points by their maximum distance to the closest prototype. One example using MNIST data is shown in Fig. 3

Our algorithm–dimensional reduction anomaly meta-algorithm (DRAMA)–consists of four main steps: (i) dimensionality reduction (*encoding*) of data to a lower-dimensional space, followed by (ii) clustering to find the main prototypes in the data, (iii) uplifting to the original space (*decoding*; optional) and finally (iv) distance measurements between the test data and the prototypes (the main clustered components) to rank potential anomalies. These steps are illustrated in Fig. 1 and discussed in turn in the following.

### Dimensionality reduction

This is the first step of the DRAMA procedure. Assume $\mathbf{X }$ is a $n_f$-dimensional feature vector. Dimensionality reduction translates $\mathbf{X }$ into another vector $\mathbf{z }$ in an *m*-dimensional space, where $m \ll n$. Good reductions keep as much important information as possible while removing noise and irrelevant information, efficiently *encoding* the data. In general, the reduction will result in loss of information but is very useful when one wants to work with or visualize the data in lower dimensions or attempts to combat the curse of dimensionality. The inverse process of lifting back up to the original space, i.e., going from $\mathbf{z }$ to $\mathbf{X }$ will be referred to as *decoding* hereafter. DRAMA uses both encoding and decoding, with primary component extraction performed in between.

The current version of DRAMA comes with 5 built-in Dimensionality Reduction Techniques (DRT)^6^:

- *Independent Component Analysis (ICA)*
  ICA is designed to separate data into a linear combination of statistically independent and non-Gaussian sources [26]. Mathematically the problem can be formulated as $x=A s$, where *x* is the data, *s* represents the independent sources, and *A* is the transformation matrix exhibiting the linear combinations [25, 38]. The advantage of ICA is that it finds maximally independent components even if the sources are not independent. The main limit of ICA is that the non-Gaussian source assumption also leads to the inability to separate Gaussian sources.
- *Non-negative Matrix factorization (NMF)*
  NMF allows the extraction of sparse and interpretable factors [19], revealing hidden features in datasets without any prior knowledge about their mean and variance [17, 20]. NMF finds two positive matrices, ${\mathbf {W}}_{d \times r}$, ${\mathbf {H}}_{r \times f}$, such that their product closely reproduces the original data matrix, ${\mathbf {W}}_{d\times f}$
  1 $$\begin{aligned} {\mathbf {W}} \approx {\mathbf {W}} {\mathbf {H}} \end{aligned}$$
  This leads to automated clustering of the data, with ${\mathbf {H}}$ indicating cluster membership and ${\mathbf {W}}$ giving the centroids of the clusters. The main disadvantage of NMF is that it does not give a unique solution [5].
- *Autoencoders (AE)*
  AE are neural network models for data compression [22]. Unlike linear algorithms (for example PCA), they can approximate highly nonlinear transformations depending on the neural network layers used. AE are successful at denoising and feature extraction [11]. In the most general application, AE tries to reconstruct the input signal after it has been forced through a tight bottleneck. This requires the network to learn the features that capture the majority of the variability of the input signal. The bottleneck represents the latent layer whose dimension is lower than the original signal. The trained encoder is a neural network which converts $\mathbf{X }$ to $\mathbf{z }$ and can be used as DRT.
- *Variational Autoencoders (VAE)*
  VAE is introduced in 2013 [30, 47] as an idea for complex generative models. Like AE they also consist of an encoder, decoder, and a loss function, but the key difference between VAE and AE is that instead of learning just a function, VAE learns the parameters of an assumed probability distribution that describes the latent variables. This is what allows it to be used as a generative model. The loss typically includes two terms like
  2 $$\begin{aligned} \varLambda =\varLambda _{\text{ recon }}+KL(q_{\text{ encoder }} (\mathbf{z } \vert \mathbf{X }),p(\mathbf{z })) \end{aligned}$$
  where *KL* is the Kullback–Leibler divergence. The first term is the reconstruction loss, and the second term is a regularizer that measures how much the encoder $q_{\text{ encoder } }(\mathbf{z } \vert \mathbf{X })$ is similar to the assumed prior distribution $p(\mathbf{z })$. In VAE $p(\mathbf{z })$ is typically taken to be a zero mean and unit variance normal distribution. For more info see, e.g., [7].
- *Principal Component Analysis (PCA) *
  One of the simplest and most common DRT is principal component analysis (PCA). PCA creates a new basis of the most important *m* linear combinations of the data through covariance matrix diagonalization [23, 41]. Since PCA is a linear mapping it fails to capture any nonlinear relationships in the data.

DRAMA uses ICA, NMF, AE, VAE, and PCA as the possible dimensionality reduction techniques, though user-supplied DRT can easily be included. These DRT reduce the input features to low dimensions as a preprocessing step for clustering and extraction of the prototype components.

### Prototype extraction

In this section, the second and third steps in DRAMA are explained, prototypes and how they can be extracted. Having encoded the features down to a low-dimensional latent space (here $\mathrm{D}=2$) using a choice of DRT, we can efficiently perform unsupervised clustering to detect clusters.

**Fig. 2:**
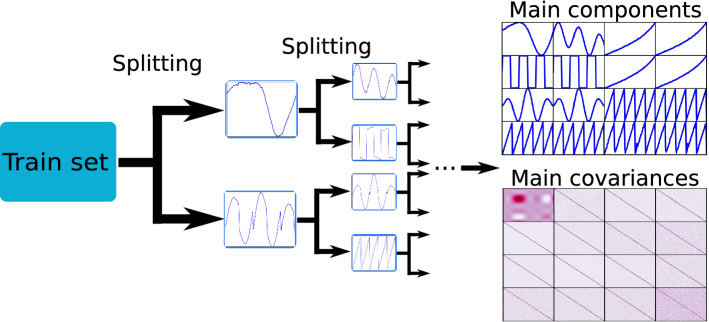
Illustration of the prototype extraction process in DRAMA. Given a chosen depth (in this case $n_s = 3$), clustering/splitting iteratively extracts more detailed information about the different shapes in the data to use as prototypes

As illustrated in Fig. 2, the data are hierarchically split into clusters. Initially, the data are split into two clusters and then successively split until after $n_s$ steps, there are $2^{n_s}$ detected prototypes, ${\mathcal {C}}=\{P_{1},P_{2}, \dots P_{2^{n_s}}\}$. There is no concern that the $2^{n_s}$ clusters represent true subclasses; $n_s$ is simply a hyperparameter designed to find anomalies. While smaller $n_s$ may cause more false positives, larger $n_s$ may allow masking of anomalies (since they happen to be close to some prototype) and hence lead to false negatives. Having found the $2^{n_s}$ clusters one can select the center^7^ of each cluster and can now choose whether or not to decode it to the original, high-dimensional, feature space. Empirically we find that decoding to the original space gives better results. Either way there are $2^{n_s}$ prototypical components representative of the “average” (inlier) data.

**Fig. 3:**
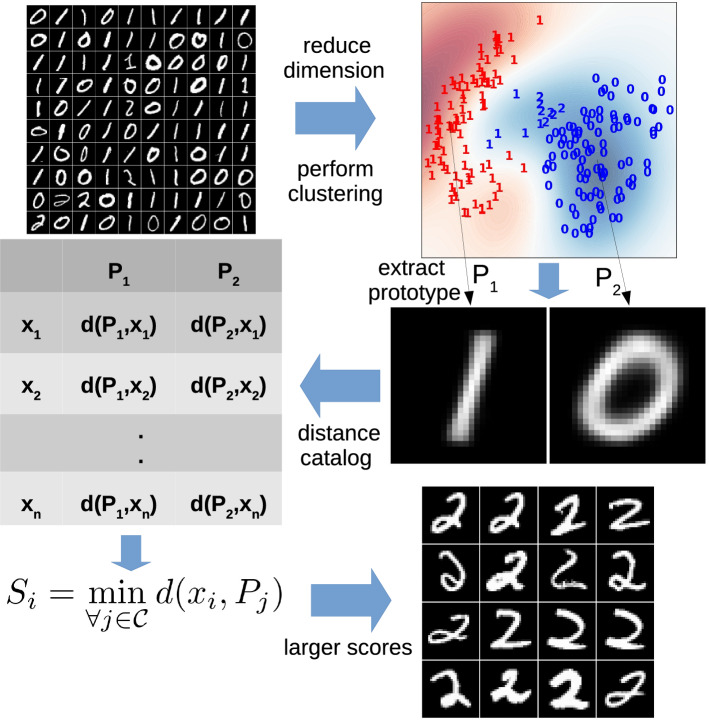
This figure shows how DRAMA finds outliers in a subset of MNIST handwritten digits data. As a simple challenge, two digits (0 and 1) are assumed to be inliers and one digit (2) is assumed as anomalous data points. Notice in the bottom right how DRAMA pulls out the many 2’s (the anomalies in this example)

### Identifying anomalies

Having decoded the prototypes, the next step is computing the distance between them and each test data point. It is possible to compute the distances using encoded prototypes as well but we find the results are better while using the decoded prototypes. This requires a distance metric (hereafter “metric”), $d(x_i, P_j )$, where $x_i$ is *i*th data point and $P_j$ is the *j*th prototype. For any choice of metric, the predicted anomalies are then ranked by $S_i$ where

3 $$\begin{aligned} S_i = \min _{\forall j \in {\mathcal {C}}} d(x_i,P_j) \end{aligned}$$

Considering the above equation, the choice of metric is another flexibility of DRAMA. Currently, DRAMA includes nine different metrics: Cityblock (L1), L2, L4, as well as the inverse variance weighted L2 and L4 distance metrics, Bray–Curtis (BC), Chebyshev [9], Canberra [31], correlation [51] and Mahalanobis. These metric definitions are given in Table 1. The minimum distance between a test data point and the prototypes shows how much a given data point is anomalous.

**Table 1:** Metric options available in DRAMA: *d*(*u*, *v*) is the distance between data points *u* and *v*; $\bar{u}$ and $\bar{v}$ are the averages of *u* and *v*, respectively

Metric	Definition
L1	$\sum _i {\left| u_i - v_i \right| }$
L2	${||u-v||}_2$
L4	${||u-v||}_4$
wL2	${||\frac{u-v}{\sigma }||}_2$
wL4	${||\frac{u-v}{\sigma }||}_4$
Bray–Curtis	$\sum {|u_i-v_i|} / \sum {|u_i+v_i|}$
Chebyshev	$\max _i {|u_i-v_i|}$
Canberra	$\sum _i \frac{|u_i-v_i|}{|u_i|+|v_i|}$
Correlation	$1 - \frac{(u - \bar{u}) \cdot (v - \bar{v})}{{||(u - \bar{u})||}_2 {||(v - \bar{v})||}_2}$
Mahalanobis	$\sqrt{ (u-v) C^{-1} (u-v)^T }$

Figure 3 shows DRAMA workflow on a hypothetical challenge made out of the MNIST dataset^8^.

## Datasets for testing DRAMA

Considering the NFL theorems, it is always possible to find or simulate a dataset that suites or completely confuses any specific algorithm. It is therefore important to examine the performance of DRAMA in a variety of different challenges. Three simulated anomaly detection challenges are designed for this purpose and then several blind-selected real-world anomaly benchmarks are used to evaluate DRAMA.

### Simulated challenges

**Fig. 4:**
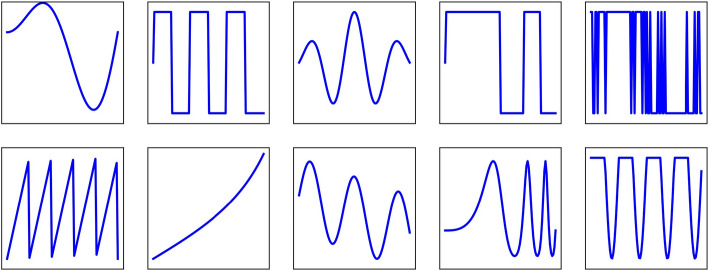
The ten base classes used as the simulated classes in all three of the synthetic time series challenges. Noise and a variety of anomalies were then added on top of these base classes to form the input data

There is an infinite number of ways to simulate inliers and anomalies. In this work 10 different classes of continuous “time series” are considered, shown in Fig. 4, representing a wide range of behaviors one might find in the real world.

**Fig. 5:**
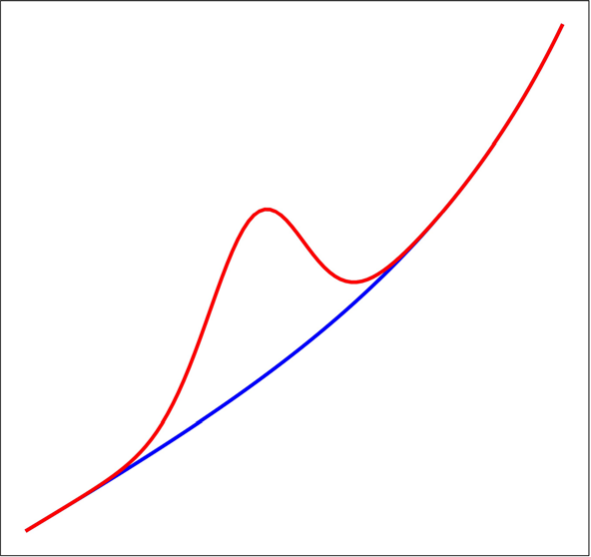
Example of an event anomaly used in Challenge-I. The blue curve is an inlier while the red curve shows a compact, anomalous event

These 10 shapes are perturbed by random Gaussian noise and random scaling in both *x* and *y* directions. Then, there are two anomaly detection challenges, each with two sub-challenges which differ only in the dimensionality of the data and one other challenge to evaluate how DRAMA can deal with multi-class outliers and discriminate between different anomalous classes after the detection phase. The details of the challenges are explained in more detail as follows:

- *Challenge-I: Compact Anomalies*
  In the first challenge, compact Gaussian “bump” anomalies are added to the 10 classes with a random location. The bump amplitude is chosen in the range $0.3-0.4$ while the width is chosen from $0.08-0.1$. There are 1000 inliers and 50 anomalies for each chosen shape. Finally, noise is produced from a zero-mean Gaussian with $\sigma =0.3$, comparable to the anomaly amplitude. This task is broken into two sub-challenges, labeled **C-Ia** and **C-Ib**, which differ only in the dimensionality of the data. For **C-Ia (C-Ib)**, $n_f = 100\, (3000)$ are chosen, respectively. An example of an event anomaly is shown in Fig. 5.
- *Challenge-II*
  The second challenge uses 9 of the shapes in Fig. 4 to produce 500 inliers while the remaining shape is used to produce 50 anomalies. To be more robust, the choice of the class used for the anomalies is permuted and the averaged result is reported. Uncorrelated Gaussian noise ($\mu =0$, $\sigma = 0.8$) is added in all cases. As before, this challenge is split into two sub-challenges, labeled **C-IIa** and **C-IIb**, which again differ only in the dimensionality of the data with $n_f = 100$ and 3000, respectively.
- *Challenge-III*
  In the third challenge (**C-III**), it is assumed that there are underpopulated classes in the dataset. The underpopulated classes occur frequently enough to be considered inliers but they are relatively rare in comparison with other classes. This represents, e.g., some phenomenon that is already discovered but not very common.
  To explain the third challenge, let us assume that we are fully aware of the existing classes in the training set (either large or small classes). This is supplemented by new observations that include both the known classes and outliers. This condition might make a problem for usual anomaly detection methods and requires data augmentation techniques since there are underpopulated classes/unbalanced training data.
  In the third challenge, a similar situation is simulated; but this time there is more than one class of outliers in the dataset and one wishes to classify them into multiple classes.
  **C-III** is designed in a way that the inlier dataset (“training data”) is highly unbalanced in term of subclasses. It includes $4\times 2000$ data points inherited of four of the ten base shapes shown in Fig. 4 and $4\times 50$ data points drawn from another four shapes with $n_f=100$, leading to a total of $4\times 2000+4\times 50=8200$ inliers instances. The test set includes a similar distribution of inliers and also 50 outliers drawn from each of the remaining 2 shapes. Hence the test set contains 8200 inliers and 100 outliers.

### Real datasets

In this study, 20 real-world datasets^9^ are chosen at random from the ODSS^10^ database. This is a standard testbed for outlier detection and has been used in many earlier works, including [2, 46, 50]. A summary of the different datasets used, including the dimensionality of the data and number of inliers and outliers, is shown in Table 2.

**Table 2:** Real-world dataset summary from the ODSS Benchmark showing the number of examples, the dimensionality of the feature set, and the number of outliers in each dataset

Dataset	# points	# dim.	# outliers
Lympho	148	18	6
Breastw	683	9	239
Wine	129	13	10
Vertebral	240	6	30
Glass	214	9	9
Pima	768	8	268
Thyroid	3772	6	93
Ionosphere	351	33	126
Cardio	1831	21	176
wbc	378	30	21
Arrhythmia	452	274	66
Vowels	1456	12	50
Satellite	6435	36	2036
Satimage-2	5803	36	71
Optdigits	5216	64	150
Mammography	11183	6	260
Shuttle	49097	9	3511
Mnist	7603	100	700
Pendigits	6870	16	156
Musk	3062	166	97

### Scoring metrics

To test the robustness, all the algorithms are run 10 times on each test dataset. We then report the average performance for three relevant score metrics suited for anomaly detection, namely: area under the ROC Curve (AUC), Matthews correlation coefficient (MCC) and rank-weighted score (RWS) [48].

The MCC lies between $-1$ and 1[37] and is defined by

4 $$\begin{aligned} \text{ MCC } = \frac{TP \times TN - FP \times FN}{(TP+FP)(TN+FN)(TP+FN)(TN+FP)} \end{aligned}$$

where *TP*(*TN*) and *FP*(*FN*) are the True Positive (Negative) and False Positive (Negative) ratios [37]. Perfect predictions yield MCC$=1$.

Given a ranked list of length *N* of the most likely outliers, the RWS is defined by

5 $$\begin{aligned} \text{ RWS } = \frac{1}{N(N+1)}\sum _{i=1}^N w_i I_i \end{aligned}$$

where the weight $w_i \equiv N + 1 - i$ and is large if *i* is small and decreases to unity for $i = N$. Here, $I_i$ is an indicator function which is unity if the *i*th object is an outlier and 0 otherwise and the sum is over the top *N* anomaly candidates. Here, *N* represents the number of anomalies. The RWS rewards algorithms whose anomaly scores correlate well with the true probability of being an anomaly. Although the performance of the algorithms in terms of the three metrics is similar, they differ when the feature space dimension is small.

## Results and discussion

DRAMA is, by design, very flexible, since it is composed of a large number of related algorithms, differing by choice of DRT, clustering, distance and scoring metrics. As a result, a DRAMA algorithm beats LOF and i-Forest on every simulated data challenge and 17 out of 20 real-world challenges in terms of AUC. Considering the problems that are investigated in this study, the Cityblock metric, and AE & NMF DRTs are the most successful on average. Because of the NFL theorems, DRAMA’s superiority cannot hold in general of course, but the results show that if one DRT or metric does not perform well, another one likely will.

**Fig. 6:**
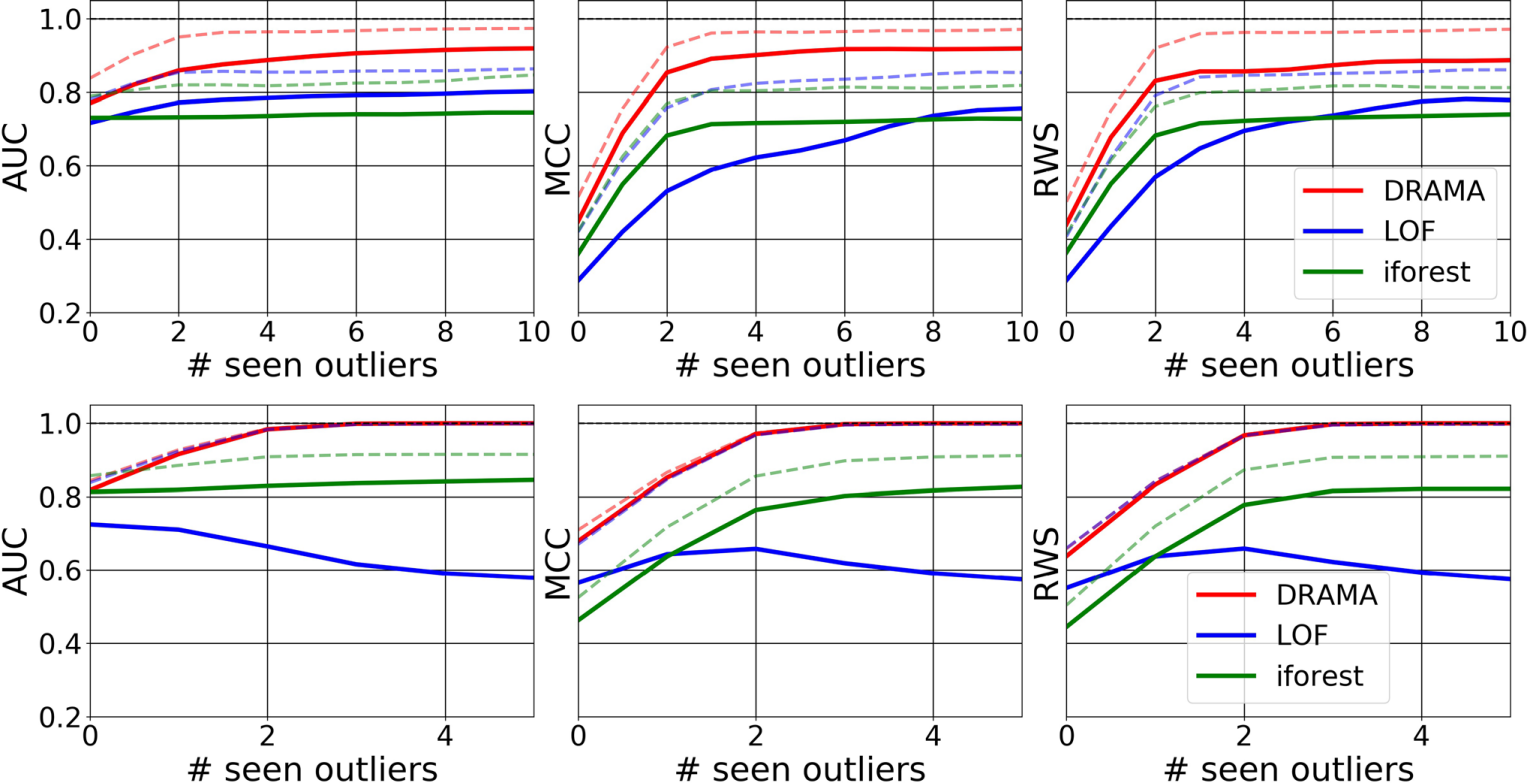
Performance in the compact anomaly challenges, for $n_f=100$ (top, **C-Ia**) and $n_f=3000$ (bottom, **C-Ib**), as a function of the number of seen anomalies. The solid line is the average performance while the dashed line is the maximum performance. DRAMA is far superior to both other algorithms. In particular, in high dimensions and for AUC, DRAMA is the only one of the algorithm that improves as it sees more outlier examples

**Fig. 7:**
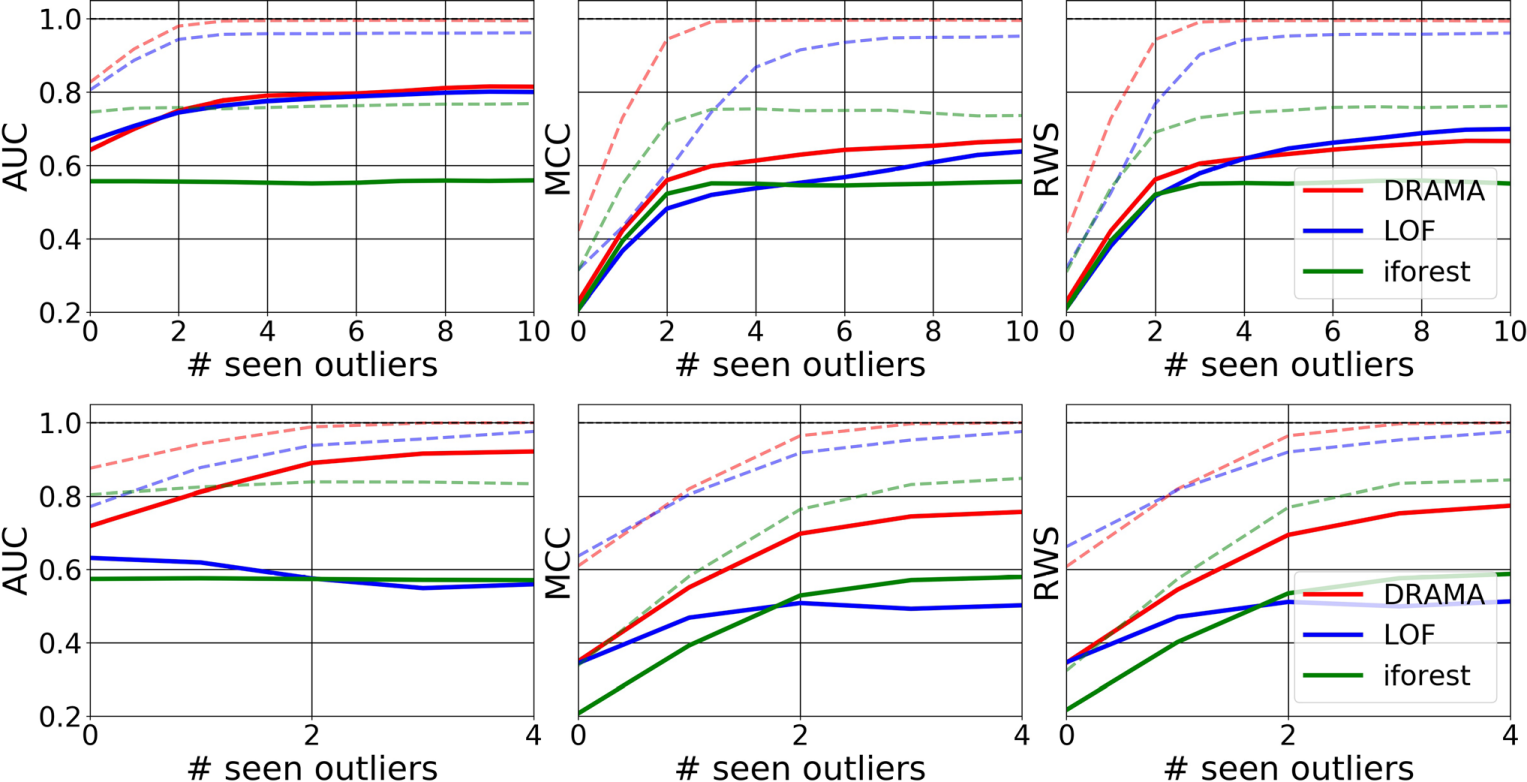
Performance in the second challenge, for $n_f=100$ (top, **C-IIa**) and $n_f=3000$ (bottom, **C-IIb**), as a function of the number of seen anomalies. The solid line shows the average performance and the dashed line the maximum performance. DRAMA is again equal to, or superior to, both LOF and iForest. DRAMA particularly shines in the high-dimensional case

**Fig. 8:**
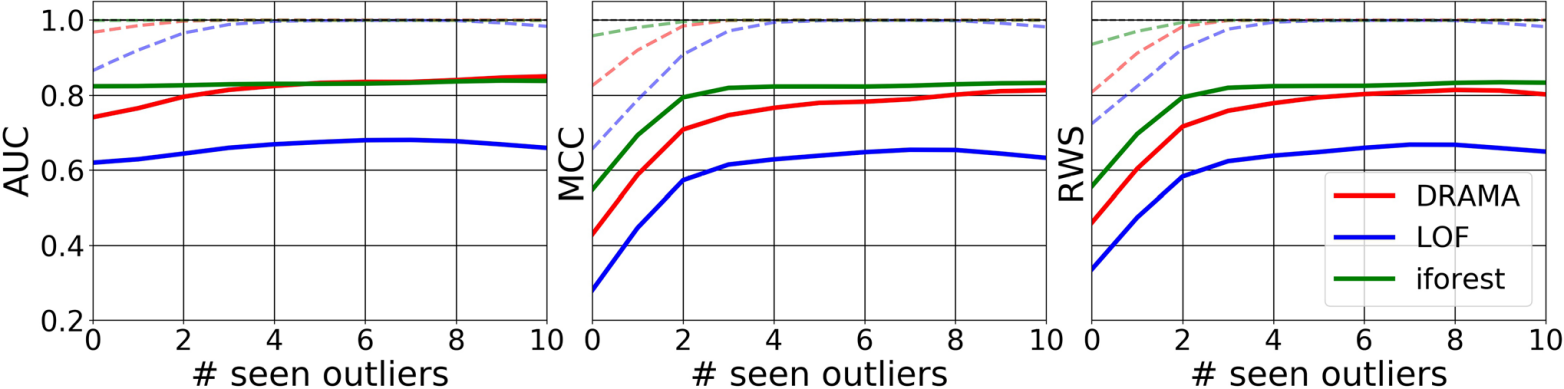
Average (solid lines) and best (dashed lines) performance on the 20 real datasets where both iForest and DRAMA outperform LOF. Although DRAMA is not as good as iForest on average in this case, note that all the datasets have dimensionality less than 300 and hence are not ideally suited to DRAMA

The flexibility of the DRAMA framework is particularly useful when one has seen a few anomalies or outliers. In this case, it is possible to learn the best DRT-clustering-metric combination to enable optimal detection of the anomalies for the chosen figure of merit. To illustrate this capability, we give DRAMA, LOF, and i-Forest the ability to learn from a variable number of seen anomalies/outliers. This is used to select the best configuration of hyperparameters for all the algorithms to maximize the figure of merit (AUC, MCC, RWS). The results for the simulated challenges are shown in Figs. 6, 7, and Table 3, and for the real datasets in Fig. 8. The results show both the mean and best results over 10 runs for each of the challenges and each algorithm.

While LOF and i-Forest are competitive on the simulated challenges in low dimensions ($n_f = 100$) DRAMA particularly shines in high dimensions ($n_f = 3000$). On the real-world datasets, we considered which have small numbers of points and dimensions $< 300$ the performance of DRAMA and i-Forest are comparable and significantly better than LOF. In terms of time consumption performance, DRAMA is slightly faster than LOF and iForest, averaging 0.33*s* per run over a range of experiments and tests.

**Table 3:** Results for Challenge-III using ICA as dimensionality reduction transformation and Mahalanobis as the metric. In this challenge, there are four rare classes in the training data that may confuse the algorithms and two outlier classes not present in the test data. The final column gives the average precision DRAMA achieves in classifying the outliers into these two classes, something LOF and i-Forest cannot do at all

AUC(%)			MCC(%)			RWS(%)			Precision(%)
DRAMA	LOF	i-Forest	DRAMA	LOF	i-Forest	DRAMA	LOF	i-Forest	DRAMA
$\mathbf {100}$	$73\pm 17$	$78\pm 12$	$99\pm 1$	$20\pm 27$	$25\pm 26$	$\mathbf {99\pm 2}$	$22\pm 22$	$25\pm 21$	$\mathbf {99\pm 1}$

The best results are in bold style

In Challenge III, there are two complications: (1) there are four rare classes that are present in both the training and test data and are red-herrings for the algorithms, and (2) there are two different classes of outliers, present only in the test set.

Here, DRAMA completely outperforms the other algorithms, achieving almost perfect performance, see Table 3, while both LOF and iForest perform very poorly in terms of MCC and RWS. Beyond this, DRAMA is able to cluster the outlier examples into classes by using their distances from the inlier prototypes. DRAMA classifies the outliers into the correct classes almost perfectly, achieving a precision of $99\pm 1 \%$. Neither LOF nor iForest can classify outliers into different classes by default.

However, because of the no free lunch theorems, no anomaly detection algorithm can outperform all other algorithms on all datasets and for all metrics. We see this in, for example, the performance of DRAMA on the wbc and satellite datasets that was poorer than LOF and/or i-Forest for some metrics. This is further illustrated in Fig. 8 which shows that, averaged over multiple runs, DRAMA performs slightly worse than i-Forest on the low-dimensional real-world datasets (though better than LOF).

## Conclusion

We present DRAMA, a general python package for anomaly detection that uses dimensionality reduction and unsupervised clustering to identify prototypes. The distance to the prototypes then leads to the detection of the potential anomalies and can spontaneously cluster outliers into subclasses. All aspects of this workflow are flexible, making DRAMA attractive in the supervised/online anomaly detection, where there are known outliers which can be used to optimize discrete and continuous hyperparameter combinations. DRAMA’s accuracy is evaluated against the commonly used algorithms isolation forest (i-Forest) and local outlier factor (LOF), using a wide variety of simulated and real datasets in up to 3000 dimensions. DRAMA particularly excelled on the simulated time series data, winning every challenge. On the very inhomogeneous and fairly low-dimensional real-world datasets considered in this study, DRAMA was highly competitive with LOF and i-Forest. DRAMA is capable of being used for data exploration and unsupervised classification and its flexibility allows extending evaluation metrics to include, for example, robustness and resilience [21, 54]. Finally, it will be interesting to modify and test DRAMA for novelty detection and anomaly detection in images. We leave this to future work.

## Footnotes

1 https://www.lsst.org/.

2 https://www.skatelescope.org/.

3 http://no-free-lunch.org/.

4 DRAMA is based on the popular scikit-learn [42] and TensorFlow [4] packages and comes with a Jupyter notebook interface for ease of use.

5 https://github.com/vafaei-ar/drama.

6 DRAMA is modular and easy to extend to any other DRT.

7 Here, taken to be the mean of each cluster.

8 The code for this example is shared on DRAMA repository.

9 We were limited to 20 by computational resources.

10 http://odds.cs.stonybrook.edu/.

## Declarations

### Code availability

All codes and data are released as a python package on GitHub. (https://github.com/vafaei-ar/drama).

## References

[1] Aggarwal CC, Hinneburg A, Keim DA (2001) On the surprising behavior of distance metrics in high dimensional spaces. In: ICDT, vol. 1, pp 420–434. Springer

[2] Aggarwal CC, Sathe S (2015). Theoretical foundations and algorithms for outlier ensembles. ACM SIGKDD Explor Newslett.

[3] Ali R, Khan MUK, Kyung CM (2020) Self-supervised representation learning for visual anomaly detection. arXiv preprint arXiv:2006.09654

[4] Allaire J, Eddelbuettel D, Golding N, Tang Y (2016) tensorflow: R Interface to TensorFlow. https://github.com/rstudio/tensorflow

[5] Berry MW, Browne M, Langville AN, Pauca VP, Plemmons RJ (2007). Algorithms and applications for approximate nonnegative matrix factorization. Comput Stat Data Anal.

[6] Beyer K, Goldstein J, Ramakrishnan R, Shaft U (1999) When is “nearest neighbor” meaningful? In: International conference on database theory, pp 217–235. Springer

[7] Blei DM, Kucukelbir A, McAuliffe JD (2017) Variational inference: A review for statisticians. J Am Stat Assoc (just-accepted)

[8] Breunig MM, Kriegel HP, Ng RT, Sander J (2000) Lof: identifying density-based local outliers. In: ACM sigmod record, vol. 29, pp 93–104. ACM

[9] Cantrell CD (2000). Modern mathematical methods for physicists and engineers.

[10] Carrara F, Amato G, Brombin L, Falchi F, Gennaro C (2020) Combining gans and autoencoders for efficient anomaly detection. arXiv preprint arXiv:2011.08102

[11] Chen M, Xu Z, Weinberger K, Sha F (2012) Marginalized denoising autoencoders for domain adaptation. arXiv preprint arXiv:1206.4683

[12] Dawoud A, Shahristani S, Raun C (2019) Dimensionality reduction for network anomalies detection: A deep learning approach. In: Workshops of the international conference on advanced information networking and applications, pp 957–965. Springer

[13] DuMouchel W, Schonlau M (1998) A fast computer intrusion detection algorithm based on hypothesis testing of command transition probabilities. In: KDD, pp 189–193

[14] Eiteneuer B, Hranisavljevic N, Niggemann O (2019) Dimensionality reduction and anomaly detection for cpps data using autoencoder. In: ICIT, pp 1286–1292

[15] Ester M, Kriegel HP, Sander J, Xu X (1996). A density-based algorithm for discovering clusters in large spatial databases with noise. Kdd.

[16] Fan H, Zaïane OR, Foss A, Wu J (2006) A nonparametric outlier detection for effectively discovering top-n outliers from engineering data. In: Pacific-Asia conference on knowledge discovery and data mining, pp. 557–566. Springer

[17] Févotte C, Idier J (2011). Algorithms for nonnegative matrix factorization with the $$\beta$$-divergence. Neural Comput.

[18] George A, Vidyapeetham A (2012). Anomaly detection based on machine learning: dimensionality reduction using pca and classification using svm. Int J Computer Appl.

[19] Gillis N (2014) The why and how of nonnegative matrix factorization. Regul Optim Kernels Support Vector Mach 12(257)

[20] Gillis N (2017) Introduction to nonnegative matrix factorization. arXiv preprint arXiv:1703.00663

[21] Han B, Liu C, Zhang W (2016). A method to measure the resilience of algorithm for operation management. IFAC-PapersOnLine.

[22] Hinton GE, Zemel RS (1994). Autoencoders, minimum description length and Helmholtz free energy. Adv Neural Inform Process Syst.

[23] Hotelling H (1933). Analysis of a complex of statistical variables into principal components. J Educ Psychol.

[24] Huang T, Sethu H, Kandasamy N (2016). A new approach to dimensionality reduction for anomaly detection in data traffic. IEEE Trans Netw Serv Manage.

[25] Hyvärinen A, Karhunen J, Oja E (2004). Independent component analysis.

[26] Hyvärinen A, Oja E (2000). Independent component analysis: algorithms and applications. Neural Netw.

[27] Iacus SM, Sermi F, Spyratos S, Tarchi D, Vespe M (2020) Anomaly detection of mobile positioning data with applications to covid-19 situational awareness. arXiv preprint arXiv:2011.04356 10.1007/s42081-021-00109-z PMC7934126 35425884

[28] Juvonen A, Hamalainen T (2014) An efficient network log anomaly detection system using random projection dimensionality reduction. In: 2014 6th international conference on new technologies, mobility and security (NTMS), pp 1–5. IEEE

[29] Juvonen A, Sipola T, Hämäläinen T (2015). Online anomaly detection using dimensionality reduction techniques for http log analysis. Comput Netw.

[30] Kingma DP, Welling M (2013) Auto-encoding variational bayes. arXiv preprint arXiv:1312.6114

[31] Lance GN, Williams WT (1966). Computer programs for hierarchical polythetic classification (“similarity analyses’). Comput J.

[32] León C (2020). Detecting anomalous payments networks: A dimensionality-reduction approach. Latin Am J Central Banking.

[33] Li JW, Zhang W, Yang G, Tu S, Chen X (2009). Thermal-error modeling for complex physical systems: the-state-of-arts review. Int J Adv Manufac Technol.

[34] Li Z, Xu W, Huang A, Sarrafzadeh M (2012) Dimensionality reduction for anomaly detection in electrocardiography: A manifold approach. In: 2012 ninth international conference on wearable and implantable body sensor networks, pp 161–165. IEEE

[35] Lin HW, Tegmark M, Rolnick D (2017). Why does deep and cheap learning work so well?. J Stat Phys.

[36] Liu FT, Ting KM, Zhou ZH (2008) Isolation forest. In: Data Mining, 2008. ICDM’08. Eighth IEEE international conference on, pp 413–422. IEEE

[37] Matthews BW (1975). Comparison of the predicted and observed secondary structure of t4 phage lysozyme. Biochimica et Biophysica Acta (BBA)-Protein Struct.

[38] Naik GR (2012) Introduction: independent component analysis. In: Naik GR (ed) Independent component analysis for audio and biosignal applications. InTech

[39] Naud L, Lavin A (2020) Manifolds for unsupervised visual anomaly detection. arXiv preprint arXiv:2006.11364

[40] Pang G, Shen C, Cao L, Hengel Avd (2020) Deep learning for anomaly detection: A review. arXiv preprint arXiv:2007.02500

[41] Pearson K (1901). Liii. on lines and planes of closest fit to systems of points in space. The London, Edinburgh, Dublin Philos Mag J Sci.

[42] Pedregosa F, Varoquaux G, Gramfort A, Michel V, Thirion B, Grisel O, Blondel M, Prettenhofer P, Weiss R, Dubourg V, Vanderplas J, Passos A, Cournapeau D, Brucher M, Perrot M, Duchesnay E (2011). Scikit-learn: Machine learning in Python. J Mach Learn Res.

[43] Pham T, Lee S (2016) Anomaly detection in the bitcoin system-a network perspective. arXiv preprint arXiv:1611.03942

[44] Pol AA, Berger V, Germain C, Cerminara G, Pierini M (2019) Anomaly detection with conditional variational autoencoders. In: 2019 18th IEEE international conference on machine learning and applications (ICMLA), pp. 1651–1657. IEEE

[45] Ramaswamy S, Rastogi R, Shim K (2000) Efficient algorithms for mining outliers from large data sets. In: Proceedings of the 2000 ACM SIGMOD international conference on Management of data, pp 427–438

[46] Rayana S, Akoglu L (2016). Less is more: Building selective anomaly ensembles. ACM Trans Knowl Discovery Data (TKDD).

[47] Rezende DJ, Mohamed S, Wierstra D (2014) Stochastic backpropagation and approximate inference in deep generative models. arXiv preprint arXiv:1401.4082

[48] Roberts E, Bassett BA, Lochner M (2019) Bayesian anomaly detection and classification. arXiv preprint arXiv:1902.08627

[49] Sakurada M, Yairi T (2014) Anomaly detection using autoencoders with nonlinear dimensionality reduction. In: Proceedings of the MLSDA 2014 2nd workshop on machine learning for sensory data analysis, pp 4–11

[50] Sathe S, Aggarwal C (2016) Lodes: Local density meets spectral outlier detection. In: Proceedings of the 2016 SIAM international conference on data mining, pp 171–179. SIAM

[51] Székely GJ, Rizzo ML, Bakirov NK (2007). Measuring and testing dependence by correlation of distances. Ann Stat.

[52] Thudumu S, Branch P, Jin J, Singh JJ (2020). A comprehensive survey of anomaly detection techniques for high dimensional big data. J Big Data.

[53] Williams G, Huang Z (1997) Mining the knowledge mine. Advanced topics in artificial intelligence pp 340–348

[54] Zhang W, Van Luttervelt C (2011). Toward a resilient manufacturing system. CIRP Ann.

